# Molecular Distinctions, Diagnosis, and Mechanism-Based Therapies in Lipedema and Obesity

**DOI:** 10.3390/cimb48090892

**Published:** 2026-09-01

**Authors:** Yiğit Ege Güney, Sıla Çağla Demiralay, İlke Keser

**Affiliations:** 1 Department of Occupational Therapy, Faculty of Health Sciences, Istanbul Topkapi University, Istanbul 34087, Türkiye; yigitegeguney@topkapi.edu.tr; 2 Institute of Health Sciences, Gazi University, Ankara 06540, Türkiye; 25854402002@gazi.edu.tr; 3 Department of Physiotherapy and Rehabilitation, Faculty of Health Sciences, Gazi University, Ankara 06490, Türkiye

**Keywords:** lipedema, obesity, adipose tissue disorders, pathophysiology, estrogen, inflammation, lymphatic system, pain, targeted therapy, physical therapy

## Abstract

Lipedema and obesity are often misdiagnosed or clinically confused yet arise via distinct mechanisms, complicating diagnosis and treatment. This review synthesizes evidence differentiating these conditions across genetic, hormonal, inflammatory and mechanical pathways to identify therapeutic targets. Lipedema may involve genetic predisposition (forkhead box C2 [FOXC2], prospero homeobox 1 [PROX1]), hormonal dysregulation with aberrant aromatase activity, and altered adipogenesis (peroxisome proliferator-activated receptor gamma [PPARγ], CCAAT/enhancer-binding protein [C/EBP]). A proinflammatory microenvironment with macrophage M1/M2 imbalance, elevated interleukin-6 (IL-6) and tumor necrosis factor-alpha (TNF-α), and extracellular matrix remodeling is hypothesized to drive fibrosis. Emerging evidence implicates gut-derived endotoxemia (lipopolysaccharide [LPS]-toll-like receptor 4 [TLR4]-nuclear factor kappa-B [NF-κB]) and mechanotransduction (Yes-associated protein [YAP]/transcriptional coactivator with PDZ-binding motif [TAZ]) in adipocyte hypertrophy and treatment resistance. Obesity involves systemic metabolic dysfunction with visceral adiposity and cardiometabolic comorbidities. Lipedema patients maintain metabolic health, exhibit gluteofemoral fat distribution and experience neuropathic pain via nociceptor sensitization (transient receptor potential vanilloid 1 [TRPV1] and ankyrin 1 [TRPA1]) with central amplification. Weight-loss interventions are ineffective, necessitating targeted strategies. Promising targets include TLR4 antagonism, vascular endothelial growth factor C/vascular endothelial growth factor receptor-3 (VEGF-C/VEGFR3) modulation for lymphatic enhancement, YAP/TAZ inhibition and neuromodulators for pain. Physical therapy functions as a biological modifier targeting inflammation, lymphatic drainage and mechanotransduction. This review highlights promising but largely hypothesis-generating molecular insights and calls for validated biomarkers, rigorous clinical trials, and mechanism-based therapies. Many of the pathways discussed require further confirmation in human studies.

## 1. Introduction

Obesity and lipedema are two distinct diseases that are frequently misdiagnosed in clinical practice yet differ fundamentally in their clinical, pathological, and sensory aspects. Both conditions can lead to physical and psychosocial consequences, negatively impact quality of life, cause mobility restrictions and limit patients’ daily living activities [1,2,3]. 

Despite its high prevalence and debilitating effects on quality of life, lipedema remains largely underdiagnosed and is frequently misdiagnosed as obesity. This clinical overlap not only delays appropriate management but also subjects patients to ineffective weight-loss interventions, leading to frustration and disease progression. A growing body of evidence indicates that lipedema is driven by distinct molecular alterations—encompassing genetic predisposition, hormonal imbalances, chronic low-grade inflammation, lymphatic insufficiency, and aberrant mechanotransduction—that fundamentally separate it from systemic obesity. Elucidating these pathway-specific differences is essential to move beyond symptomatic management towards mechanism-based diagnosis and targeted therapeutics.

The remainder of this review is structured as follows: we first compare clinical definitions and epidemiology, then systematically examine genetic, hormonal, inflammatory, lymphatic, mechanotransduction, and sensory pathways in that order, followed by therapeutic implications and future directions.

## 2. Methodology

This is a narrative review aimed at synthesizing the available literature on the molecular distinctions between lipedema and obesity. No systematic search strategy or formal quality assessment was applied due to the emerging and heterogeneous nature of the field. Instead, we performed a comprehensive, critical, and selective review of peer-reviewed articles published up to 2026, prioritizing studies that provided mechanistic insights. To ensure a systematic and reproducible literature survey, we conducted supplementary structured searches in PubMed, Scopus, and Web of Science using the following keywords and Medical Subject Headings (MeSH) terms: “lipedema”, “obesity”, “adipose tissue”, “pathophysiology”, “estrogen”, “inflammation”, “lymphatic dysfunction”, “extracellular matrix”, “pain”, “mechanotransduction”, and “targeted therapy”. Reference lists of retrieved articles were also manually screened to identify additional relevant studies. After removing duplicates and screening titles and abstracts, we excluded non-English articles, case reports with fewer than two patients, editorials, and studies lacking original mechanistic or comparative clinical data. Following full-text assessment, a total of 148 publications (including original research articles, reviews, and book chapters) were deemed relevant and included in this qualitative synthesis. As a narrative review, we present a critical interpretation of the evidence, clearly differentiating well-established findings from hypothesis-generating proposals. Our goal is to present current hypotheses and evidence to raise awareness and guide future research, not to provide definitive clinical guidelines.

## 3. Clinical Overview and Epidemiology

Obesity is a chronic, multifactorial disease characterized by excessive fat accumulation in the body and is recognized as a significant global public health problem. The World Health Organization (WHO) classifies individuals with a Body Mass Index (BMI) ≥ 30 kg/m^2^ as obese [4]. Obesity is further categorized into three classes: individuals with a BMI of 30–34.9 kg/m^2^ are classified as Class I obese, those with a BMI of 35–39.9 kg/m^2^ as Class II obese and those with a BMI of 40 kg/m^2^ and above as Class III or morbidly obese [5]. Obesity is not limited to excess weight alone. It leads to many systemic effects such as metabolic disorders, inflammation, insulin resistance and cardiovascular diseases [6,7,8]. Factors such as a sedentary lifestyle, unhealthy eating habits and genetic predisposition play an important role in the development of obesity. Obesity is characterized by widespread fat accumulation in the body and progresses alongside systemic inflammation, insulin resistance and cardiovascular risk factors [9].

Lipedema is a chronic inflammatory disease, typically observed in women [10]. It is characterized by the abnormal, symmetrical accumulation of adipose tissue in the lower extremities [11,12,13,14] and is frequently sensitive to hormonal changes such as puberty, pregnancy and menopause. Lipedema is also associated with genetic predisposition [15].

**Figure 1:**
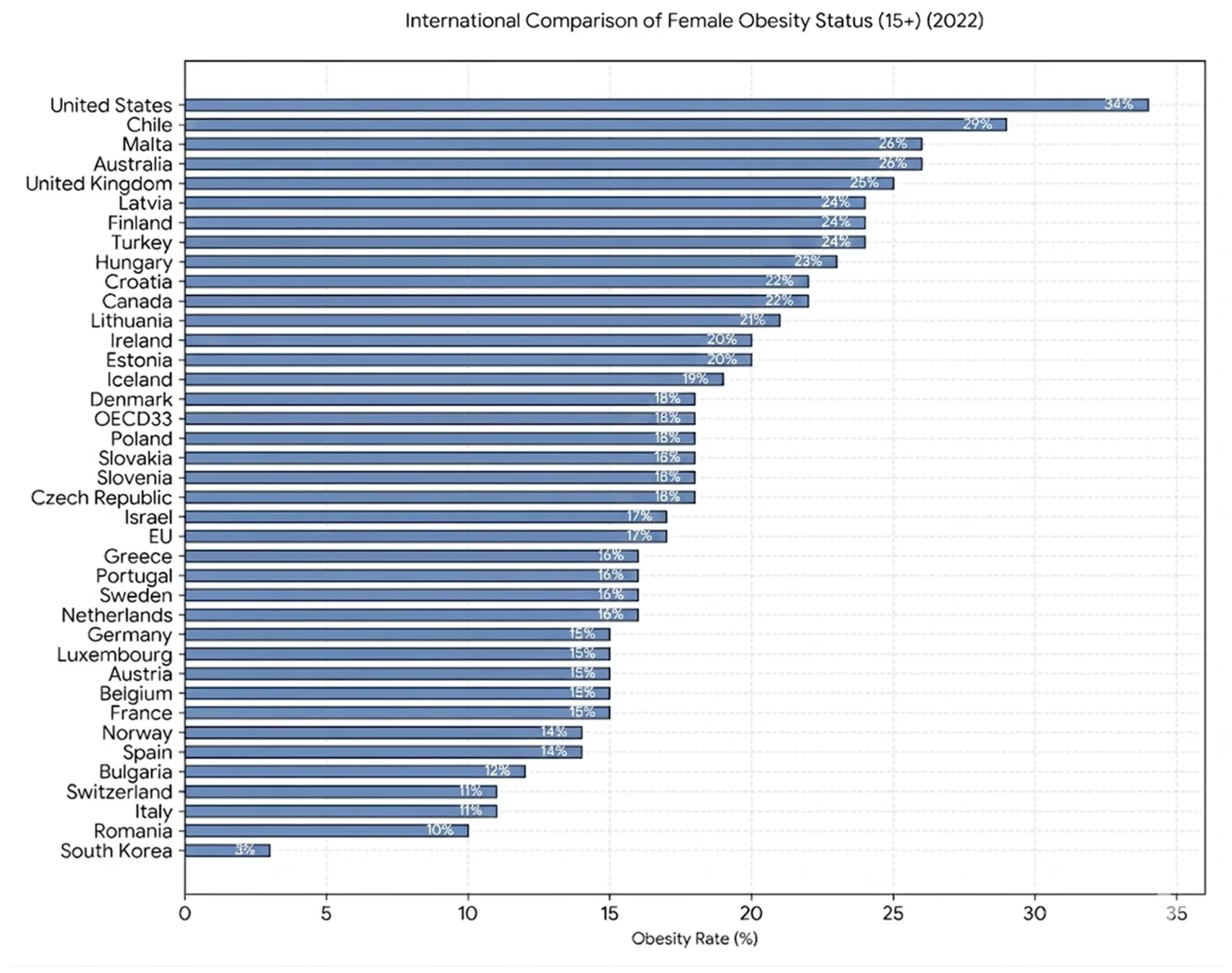
International comparison of obesity prevalence in women aged fifteen and over (OECD and EU countries) [16,17].

Obesity prevalence varies globally, with higher rates observed in women (Figure 1). Detailed country-specific data are available elsewhere [16,17]. Obesity is associated with chronic edema and lymphatic dysfunction, as highlighted by the LIMPRINT study [18].

Obesity is not limited to weight gain alone; it is also closely linked to a complex interaction of genetic predisposition [19], inflammatory processes [20,21] and environmental factors [22], as well as cardiometabolic risk factors [23,24], hormonal imbalances [25,26] and sex-based differences [27,28]. Due to its systemic effects, obesity is associated with numerous comorbid diseases [29,30,31,32]. These encompass primarily metabolic and cardiovascular diseases, but also musculoskeletal disorders [33], respiratory diseases [34,35] and psychosocial problems [36]. Epidemiological studies reveal that obesity has a strong association with comorbidities such as type 2 diabetes [37], hypertension [38], cardiovascular diseases [7,8] and osteoarthritis [39].

Although the developmental mechanism of lipedema is not fully elucidated, it is recognized as a multicomponent disease in which genetic, hormonal and vascular factors collectively play a role. The effect of estrogen in the disease process is supported by the high prevalence of lipedema in women [40]. Studies have reported a high rate of similar symptoms in the first-degree relatives of individuals diagnosed with lipedema [41,42,43]. Obesity, however, is a multifactorial condition resulting from an imbalance between energy intake and expenditure, influenced by genetic, environmental, behavioral and hormonal factors. Nonetheless, in most lipedema patients, while the region above the waist maintains a normal appearance, a marked disproportion is observed between the lower and upper extremities [44,45].

Lipedema can exhibit familial clustering, with genes related to lymphangiogenesis, such as prospero homeobox 1 (PROX1) and forkhead box C2 (FOXC2), standing out as contributing factors. Transcriptomic and proteomic data show enrichment in gene sets associated with estrogen signaling, inflammation and extracellular matrix (ECM) remodeling. Epigenetic changes and microRNA profiles are considered regulatory mechanisms in the development of lipedema [46,47].

The near-exclusive occurrence of lipedema in women points to the central role of hormonal factors, particularly estrogen, in its pathogenesis. Estrogen regulates fat distribution, expanding gluteofemoral fat depots (gynoid pattern). In individuals with lipedema, fat tissue shows higher aromatase activity, which increases the conversion of androgens into estrogen and results in elevated estrogen levels in the affected areas [48]. This elevation enhances Estrogen Receptor Alpha (ERα) signaling in adipocytes, promoting adipogenesis and the expression of lipogenic genes (e.g., lipoprotein lipase [LPL], Peroxisome proliferator-activated receptor gamma [PPARγ]). Estrogen stimulation of Vascular Endothelial Growth Factor (VEGF) expression may increase microvascular permeability, contributing to edema development. Shifts in the ERα/ERβ receptor balance and the intersection of estrogen with proinflammatory signaling pathways (NF-κB) exacerbate the disease’s inflammatory component. These processes help explain why lipedema symptoms often become more severe during periods of hormonal change such as puberty, pregnancy and menopause. This also explains why hormone replacement therapy requires cautious use [41,48,49].

Alterations in estrogen levels are suggested to play a significant role in the development of lipedema [41]. In this context, the term “pseudopregnancy” has been used in the literature to describe a state of hormonal mimicry in which non-pregnant individuals exhibit biochemical fluctuations in estrogen, progesterone, and prolactin that resemble those observed during pregnancy [50]. These hormonal shifts are known to promote adipocyte proliferation, fluid retention, and increased vascular permeability. In individuals with lipedema, such sustained or cyclical changes in sex hormone levels may support the growth of fat cells and trigger inflammatory processes, thereby contributing to disease progression [48].

**Figure 2:**
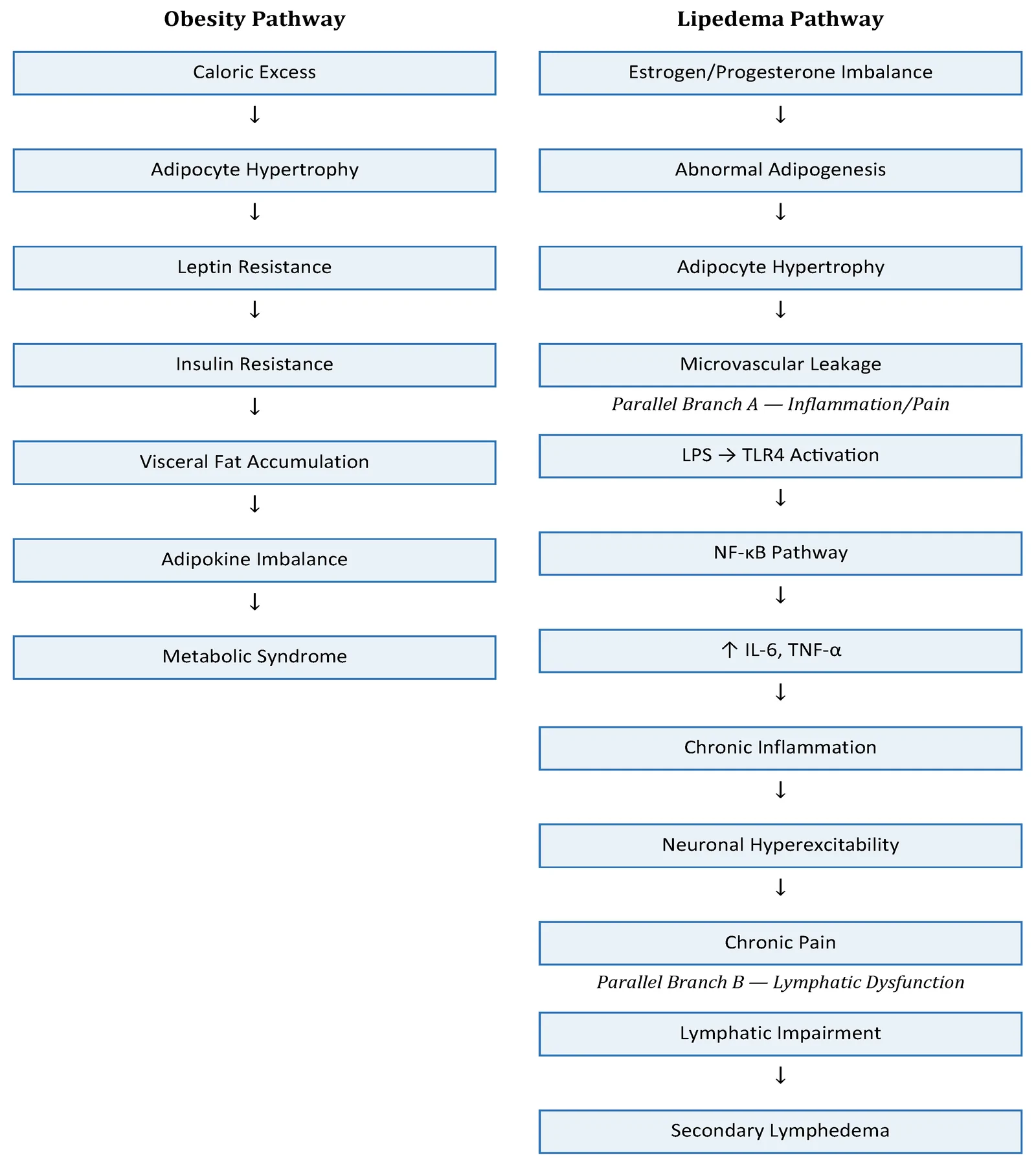
Lipedema and obesity pathways. Abbreviations: LPS: Lipopolysaccharide; TLR4: Toll-like receptor 4; NF-κB: Nuclear factor kappa-B; IL-6: Interleukin-6; TNF-α: Tumor Necrosis Factor-alpha.

In lipedema, fat accumulation occurs in specific regions and symmetrically, and typically becomes pronounced in the lower extremities [51]. Even though lipedema is common—especially in women—it is often overlooked by both patients and healthcare professionals. This lack of awareness can delay diagnosis and cause the condition to be mistaken for obesity, leading people to receive treatments that are not appropriate or effective [52,53]. Given the progressive nature of lipedema, early recognition of symptoms and the application of patient-specific therapeutic approaches can be effective in reducing disease severity and complications [14]. An overview of the key pathways involved in lipedema and obesity is provided in Figure 2.

## 4. Diagnostic Differentiation and Biomarkers

In the diagnosis and classification of lipedema, detailed clinical evaluation of the patient’s symptoms and physiological changes is of great importance [54]. Although a standardized biomarker panel and imaging methods for lipedema diagnosis are not yet established, potential biomarkers include interleukin-6 (IL-6), tumor necrosis factor-alpha (TNF-α), leptin, adiponectin and complement components. At the tissue level, techniques such as ultrasound elastography, magnetic resonance elastography and sodium (23Na) MRI assess fibrosis and sodium accumulation. Lymphatic function can be objectively measured using methods like indocyanine green near-infrared fluorescence imaging and lymphoscintigraphy.

It should be noted that the biomarkers listed above are primarily experimental and not yet validated for routine clinical use. In the presence of concomitant obesity (common in lipedema patients), the differences in these markers may diminish. Therefore, this panel should be considered as a research tool and may only be helpful in selected diagnostic dilemmas, not as a standalone diagnostic test.

The biomarker profile presented in Table 1 offers a potential framework not only for aiding differential diagnosis but also for monitoring disease progression and evaluating treatment response. For example, the relatively preserved adiponectin levels in individuals with lipedema may be interpreted as a molecular indicator of why these patients have a lower risk of metabolic complications compared to obesity. Similarly, the mild yet chronic increase in IL-6 and TNF-α, along with hyaluronan accumulation, points to potential pharmacological targets for the inflammatory and fibrotic components of the disease. In clinical practice, especially in cases of lipedema comorbid with obesity, the use of this biomarker panel could facilitate the development of personalized treatment strategies.

Lipedema is subdivided into different types based on where adipose tissue accumulates in the body. This classification is an important criterion for understanding the clinical course of the disease and determining the appropriate treatment plan. There are five subtypes.

Type I: Fat tissue increase is concentrated in the hips and gluteal region. The upper legs are less affected and deformities are not pronounced.

Type II: Hips, upper legs and the area around the knees are affected. Fat accumulation concentrates in these areas, causing an irregular appearance on the skin surface.

Type III: Widespread fat accumulation is observed across the hips and upper and lower legs. Skin dimpling and nodular formations are common.

Type IV: Fat accumulation is observed not only in the lower extremities but also in the arms. In this form, the upper body is less affected, while the arms and legs become disproportionately enlarged.

Type V: Fat accumulation becomes particularly pronounced in the lower legs. A cuff-like appearance develops at the ankles, while the feet are usually unaffected [55].

The progression of lipedema is assessed in different stages, with clinical symptoms becoming more pronounced at each stage. In the initial periods of the disease, the skin surface has a normal appearance, while in advanced stages, fibrosis, irregularity and functional losses occur in the adipose tissue. It is classified into four stages.

Stage 1: The skin maintains a smooth appearance and the adipose tissue has a soft structure. A mildly granular texture may be palpable under the skin, but no significant deformities have formed.

Stage 2: Irregular nodules develop in the adipose tissue and the skin surface takes on a mildly dimpled appearance. Patients at this stage may experience pressure sensitivity and mild pain.

Stage 3: Pronounced irregularities appear under the skin and denser nodular structures are seen in the adipose tissue. Increasing fibrotic changes can lead to tissue hardening and shape deformities in the extremities.

Stage 4: Severe edema and a presentation of secondary lymphedema may develop. Abnormal growth of adipose tissue restricts limb movement and seriously diminishes patients’ quality of life [56,57].

## 5. Pathophysiological Mechanisms

### 5.1. Adipose Tissue Perspective

Sex-based differences in obesity are also noteworthy, with women typically exhibiting peripheral (gynoid) fat accumulation, while men show more pronounced visceral (android) adiposity. These differences are associated with hormonal influences and genetic factors. Estrogen is known to have a positive effect on insulin sensitivity and the increased visceral fat in postmenopausal women contributes to the development of insulin resistance. On the other hand, androgens increase visceral adiposity in men and insulin resistance has been observed to increase in cases of hypogonadism [58,59]. Systemic inflammation, elevated free fatty acid levels and adipokine imbalances associated with obesity can negatively affect glucose metabolism, potentially accelerating the development of diabetes mellitus [60].

The endocrine system plays a critical role in the pathophysiology of obesity. Dysregulations in the hypothalamic–pituitary–adrenal axis, particularly causing elevated cortisol levels, increase central adiposity and exacerbate the severity of metabolic disorders [61,62]. Chronic low-grade inflammation is one of the fundamental processes associated with obesity. The increase in proinflammatory cytokines secreted by fat cells leads to systemic inflammation and a rise in cardiovascular disease risk [63]. This inflammatory process can impair vascular endothelial function, accelerating atherosclerotic plaque formation [64].

Adipose tissue functions not merely as an energy-storing structure but also as an active endocrine organ that drives metabolic processes [65]. While storing energy, white adipose tissue can also influence systemic metabolic processes by secreting inflammatory markers [66]. In contrast, brown adipose tissue contributes to body weight control by increasing energy expenditure through thermogenesis [67]. The reduced activity of brown adipose tissue in obese individuals decreases energy expenditure and increases the tendency for weight gain [68].

Lipedema exhibits dysregulated adipogenesis, with observations of both hyperplasia and hypertrophy in adipocytes [46,69]. The adipogenesis process involves the transcription factors PPARγ and C/EBP, with local estrogen production and aromatase activity significantly enhancing this differentiation. Unlike obesity, lipedema-affected tissue demonstrates a stronger proliferative response and a localized distribution pattern of fat. The adipokine profile is one of the key parameters distinguishing lipedema from obesity. High leptin levels and low adiponectin impact insulin sensitivity and inflammatory activity. Furthermore, a chronic inflammatory microenvironment associated with elevated IL-6 and TNF-α levels has been characterized in lipedema. ECM remodeling involves collagen accumulation, lysyl oxidase activity and matrix metalloproteinase/tissue inhibitor of metalloproteinase (MMP/TIMP) imbalance. Consequently, tissue stiffness increases, while hyaluronan and sodium storage contribute to edema and hyperexcitability of nociceptive endings [69,70].

Concurrently, individuals with lipedema exhibit impaired microvascular circulation, increased capillary permeability and interstitial fluid accumulation. These changes trigger chronic inflammatory processes, accelerating disease progression. Increased capillary permeability allows inflammatory mediators to leak into tissues, promoting edema formation and exacerbating pain and tenderness [71]. Impaired lymphatic function has been reported even in early stages, with pathways such as VEGF-C/D-VEGFR3, PROX1 and FOXC2 shown to play a role in this process. Paracrine interactions between endothelial cells, adipocytes and immune cells fuel inflammation, shaping the tissue microenvironment [72]. The inability of the lymphatic system to provide adequate drainage in this process can lay the groundwork for the development of secondary lymphedema in the advanced stages of lipedema [73].

Gluteofemoral white adipose tissue (gfWAT), located in the lower body—particularly the hip and thigh region—is a metabolically active adipose depot. This adipose tissue plays a significant role in body fat distribution and is highly sensitive to hormonal changes. In women, especially under the influence of estrogen, gfWAT is involved in energy storage and mobilization processes while also serving a critical function in regulating inflammation. This regional fat depot is metabolically active and sensitive to hormonal and inflammatory processes. gfWAT is one of the tissues most affected in lipedema, with adipocyte expansion in this area leading to inflammation and metabolic dysregulation [74]. Lipopolysaccharides (LPSs) accumulate in gluteofemoral white adipose tissue, triggering low-grade inflammation. This inflammatory process contributes to chronic pain and edema in individuals with lipedema, while simultaneously impairing insulin sensitivity and lipolysis mechanisms in adipose tissue. These alterations represent one of the significant factors that make weight loss challenging for individuals with lipedema [50,75]. Increased production of the Stearoyl-CoA desaturase-1 (SCD1) enzyme in the gluteofemoral region provides an important clue to the presence of an inflammatory environment in this area and the pathophysiology of the disease [76]. Connective tissue changes are among the characteristic features of lipedema. Irregularities in collagen synthesis can lead to reduced elasticity of the skin and subcutaneous tissues, making fat accumulation more apparent. Increased fibrous tissue formation causes the subcutaneous tissues of individuals with lipedema to gradually become harder and more nodular [77]. These changes can restrict tissue mobility in the advanced stages of lipedema, paving the way for limb deformities [78]. In addition to the structural changes in adipose tissue in lipedema, distinct immunological differences have also been defined [69]. Specifically, an increase in proinflammatory M1 macrophages and a decrease in anti-inflammatory M2 macrophages sustain the chronic inflammatory microenvironment [79]. Consequently, the release of cytokines such as TNF-α, IL-1β, and IL-6 increases and tissue inflammation can become persistent. Furthermore, an increase in mast cells and T lymphocytes suggests that lipedema progresses via immunological mechanisms distinct from those of obesity. This immunological imbalance plays a fundamental role in the emergence of characteristic findings of the disease, such as pain, edema-like complaints and fibrotic changes [80].

Lipedema and obesity are two distinct conditions that both lead to an increase in body fat, but differ in etiology, clinical presentation and pathophysiology. Lipedema is characterized by abnormal, symmetrical fat accumulation typically affecting the lower extremities. One of its most significant clinical signs is soft tissue edema, manifested by easily noticeable and generally symmetrical leg thickening and skin dimpling upon external examination [43]. Obesity, on the other hand, is defined by an elevated BMI and is recognized as a systemic metabolic disorder [81]. It involves systemic fat storage and can be linked to significant fat accumulation in the abdominal region [82].

### 5.2. Pathophysiological Perspective

Lipedema involves impairment of the lymphatic system and increased vascular permeability [72]. The role of gut health and Gut-Associated Lymphoid Tissue (GALT) is attracting increasing attention in elucidating the complex mechanisms underlying lipedema. GALT, composed of various lymphoid cells such as Peyer’s patches, lamina propria lymphocytes and intraepithelial lymphocytes located in the small intestinal mucosa and submucosa, is an important component of the immune system. While performing critical functions like nutrient absorption and defense against pathogens, this tissue is also directly affected by intestinal permeability. Intestinal permeability is controlled by tight junctions between the epithelial cells forming the gut wall. Proteins such as zonulin play a key role in regulating these tight junctions. However, gluten sensitivity, inflammatory bowel diseases, or other triggers can increase zonulin secretion, leading to the loosening of tight junctions and increased intestinal permeability. In this “leaky gut” state, lipopolysaccharides (LPS) derived from Gram-negative bacteria cross the intestinal barrier and enter systemic circulation. The activation of Myeloid Differentiation Primary Response 88 (MyD88)-dependent pathways by LPS via toll-like receptor 4 (TLR4) may lead to the translocation of nuclear factor kappa-B (NF-κB) and increased transcription of proinflammatory cytokines (TNF-α, IL-1β, IL-6). This systemic inflammatory response can create a vicious cycle by intensifying the local inflammation already present in lipedema tissue. Furthermore, LPS activation of the complement system and triggering of mast cell degranulation contribute to increased microvascular permeability, protein-rich interstitial fluid accumulation and, ultimately, the characteristic pain and edema. These findings support the idea that dietary interventions to support gut barrier integrity (probiotics, prebiotics, glutamine) and potential TLR4 antagonism should be considered as adjuvant treatment options in lipedema management [75,83].

**Figure 3:**
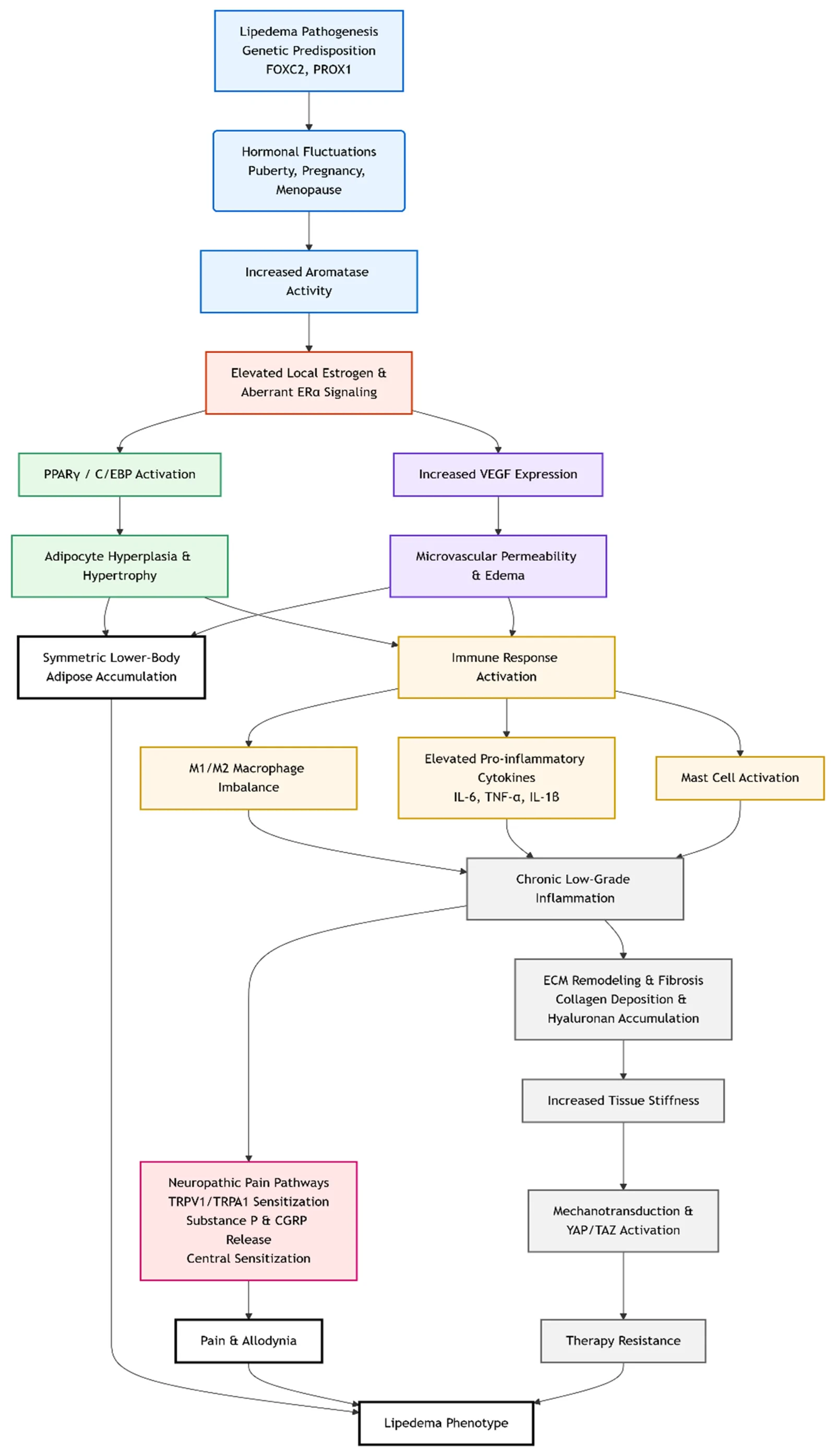
Integrated molecular pathways in the pathogenesis of lipedema, highlighting the roles of hormonal dysregulation, inflammation, lymphatic dysfunction and mechanotransduction. Abbreviations: FOXC2: Forkhead Box C2; PROX1: Prospero Homeobox 1; C/EBP: CCAAT-Enhancer-Binding Protein; VEGF: Vascular Endothelial Growth Factor; PPARγ: Peroxisome Proliferator-Activated Receptor Gamma; IL-6: Interleukin-6; TNF-α: Tumor Necrosis Factor-alpha; IL-18: Interleukin-18; TRPV1: Transient Receptor Potential Vanilloid 1; TRPA1: Transient Receptor Potential Ankyrin 1; CGRP: Calcitonin Gene-Related Peptide; YAP: Yes-Associated Protein; TAZ: Transcriptional Coactivator with PDZ-Binding Motif; ECM: Extracellular Matrix.

While complement activation and mast cell degranulation contribute to edema and pain, the imbalance in macrophage polarization (M1/M2 shift) is effective on ECM remodeling and pain sensitivity [75]. Consequently, systemic inflammation is triggered, which can further worsen the abnormal fat accumulation and lymphatic dysfunction observed in lipedema by affecting adipocyte function. Moreover, GALT function can also be negatively affected by this process. Chronic inflammation can alter the activation of immune cells and lymphokine release in GALT, which may contribute to increased permeability of lymphatic vessels and edema formation. Therefore, the balance of gut microbiota, regulation of intestinal permeability and healthy GALT function are important for the prevention and management of lipedema [84]. A simplified representation of the integrated molecular pathways including hormonal dysregulation, inflammation, lymphatic dysfunction and mechanotransduction is provided in Figure 3.

A direct mechanistic comparison reveals both shared and disease-specific pathways. For example, chronic low-grade inflammation is present in both conditions, but lipedema is characterized by a more localized M1/M2 macrophage imbalance and mast cell involvement, whereas obesity exhibits systemic metabolic inflammation driven by visceral adiposity. Hormonally, estrogen plays a dominant role in lipedema-associated adipogenesis, while obesity involves broader endocrine dysregulation (e.g., leptin resistance, cortisol excess). Lymphatic dysfunction and ECM remodeling with hyaluronan accumulation are more specific to lipedema. These distinctions underscore the need for different therapeutic approaches.

The gut microbiota is another significant factor directly associated with obesity. Disruption of the microbial balance (dysbiosis) can increase intestinal permeability, trigger inflammation and pave the way for metabolic disorders [83]. Furthermore, alterations in the production of short-chain fatty acids can directly affect appetite mechanisms and insulin sensitivity, thereby accelerating the progression of obesity [85].

### 5.3. Anthropometric Changes

The waist-to-hip ratio is a key anthropometric measure indicating abdominal fat distribution. This ratio, calculated by dividing waist circumference by hip circumference, is used to assess an individual’s metabolic health risk. One study reported that lipedema patients have less fat accumulation in the abdominal region and exhibit a more peripheral (gynoid) fat distribution. A high waist-to-hip ratio, characterized by increased visceral adiposity (abdominal obesity), is a significant risk factor for insulin resistance, cardiovascular diseases and other metabolic complications. Therefore, the lower waist-to-hip ratio observed in individuals diagnosed with lipedema suggests they may have a lower metabolic risk [86].

In obesity, since BMI alone does not reflect body fat distribution or metabolic health status, additional anthropometric measurements such as waist circumference, waist-to-hip ratio and body fat percentage also play an important role in clinical assessment [87,88].

### 5.4. Sensory Perception and Neuroplasticity

In obese individuals, an increased pain threshold, particularly to thermal and mechanical stimuli, is observed. Reduced sensitivity to thermal and mechanical stimuli has been reported in areas with dense subcutaneous adipose tissue. The abdominal region, due to excess fat tissue, shows lower sensitivity to hot and cold stimuli. This may be related to the insulating effect of subcutaneous fat or changes in transmission mechanisms resulting from mechanical pressure on nerve endings [89].

Among the possible mechanisms for these changes in sensory function, the modulation of neuroinflammation and central sensitization is significant. Chronic inflammation associated with obesity can lead to changes in the peripheral and central nervous systems, affecting sensory perception. An increase in proinflammatory cytokines can cause impaired nerve transmission and modifications in pain pathways [90]. Specifically, an increase in inflammatory mediators like leptin and IL-6 can lead to neurotransmitter imbalance in the central nervous system, altering pain perception. Changes in pain threshold, interoceptive awareness disorders and neuroinflammation have been reported in obese individuals. Hormones such as leptin and ghrelin can be effective on sensory processing in the central nervous system [91,92].

Although the pathophysiology of pain in lipedema is not fully clarified, factors such as peripheral nerve fiber damage, neuropathic processes, inflammatory response and microvascular dysfunction are thought to be involved [93]. Increased sensitivity of peripheral nociceptors (TRPV1, TRPA1, Na^+^ channels) and elevated release of neuropeptides like Substance P and Calcitonin Gene-Related Peptide (CGRP) have been reported in lipedema [94,95]. These mechanisms can increase the localized intensity of pain. At the central level, microglia and astrocyte activation leads to central sensitization, which can reinforce the experience of chronic pain [96]. In clinical assessment, palpation tenderness, pressure pain threshold and quantitative sensory tests can be used as objective measurement tools. The sensory system consists of various nerve fibers that detect mechanical, thermal and chemical stimuli from the external world and transmit them to the central nervous system [97]. In individuals diagnosed with lipedema, changes in the function of A-delta and C fibers, in particular, can be observed [98]. A-delta fibers, due to their myelinated structure, provide fast transmission, carrying sharp and localized pain. On the other hand, unmyelinated C fibers play a role in carrying burning and continuous pain sensations. Clinical observations indicate that in lipedema patients, chronic pain syndromes develop as a result of excessive C-fiber activation. Additionally, inflammation-induced neuropathic processes can increase the sensitivity of A-delta fibers, causing even light tactile stimuli to be perceived as painful [99,100].

The majority of lipedema patients report increased mechanical sensitivity in affected areas and discomfort even with light touch. These sensory changes, which are particularly pronounced in the lower extremities, lead to a decreased pressure pain threshold and increased hyperreactivity to mechanical stimuli [94]. These changes reveal that lipedema is a disease that also affects the somatosensory system.

Synaptic plasticity changes occurring in the spinal cord dorsal horn can lead to pain perception being felt more intensely and for longer durations in the sensory cortex [101]. The persistence of chronic pain can be associated with mechanisms such as increased N-Methyl-D-Aspartate (NMDA) receptor activity, decreased inhibitory signals and strengthened glutamatergic transmission [102]. These changes can lead to increased sensory sensitivity in individuals with lipedema and cause even mild stimuli to be perceived as excessively painful.

The contribution of inflammation to the pain mechanism in lipedema is considered an important component of the pathological process. Furthermore, inflammation-induced microvascular changes lead to increased capillary permeability and disrupted lymphatic flow, causing edema accumulation in tissues [103]. As edema increases, the mechanical pressure applied to nerve fibers also rises, which can lead to the worsening of sensory disorders and pain.

Sensory impairments in lipedema are pronounced, with patients experiencing chronic pain, allodynia and hyperalgesia. Tissue sensitivity can be associated with nerve fibers being under mechanical pressure. Proprioceptive dysfunction can lead to impairments in body awareness. Although there is no evidence of adipocyte enlargement in the early stages of lipedema, in advanced stages, changes in tissue morphology and increased permeability in blood vessels can cause patients’ sensory symptoms to intensify and adipocyte hyperplasia is dominant [70]. In obesity, however, hypertrophy is predominant [65]; this difference is the fundamental mechanism explaining treatment resistance [104].

## 6. Therapeutic Implications

This table can aid in determining the correct diagnosis and treatment approach by summarizing the distinguishing characteristics of lipedema and obesity (Table 2).

The management of obesity may require individualized nutrition plans, aerobic exercise programs, behavioral therapies and, in some cases, surgical interventions, whereas complete decongestive physiotherapy is effective in the treatment of lipedema [105].

The majority of patients with lipedema report significant tenderness, particularly in the lower extremities, ecchymosis due to increased capillary fragility and chronic pain. These symptoms are among the important signs that help distinguish lipedema from obesity [51]. One study measured capillary fragility using a vacuum suction method to differentiate lipedema from obesity, finding a significantly higher number of petechiae (small hemorrhages) on the skin of lipedema patients compared to obese individuals [106]. From a pathophysiological perspective, lipedema involves impairment of the lymphatic system and increased vascular permeability [72]. Especially in the advanced stages of the disease, the increasingly hardening and fibrous structure of the adipose tissue further complicates the management of lipedema. In the initial stages of lipedema, the skin surface is smooth and the subcutaneous adipose tissue shows a homogeneous distribution. However, as the disease progresses, an increase in the size and number of adipocytes occurs [51]. This process leads to increased perivascular inflammation and the dysregulation of interstitial fluid due to structural changes in the vessel walls. Consequently, intertissue edema develops, creating both mechanical pressure and increasing inflammatory cell infiltration [107]. With disease progression, structural changes in the subcutaneous adipose tissue lead to the formation of nodules. Simultaneously, increased dermal fibrosis and remodeling of the extracellular matrix cause tissue stiffness to become pronounced. This process manifests as skin dimpling and increased sensitivity to touch. Adipocyte hypertrophy and fibrosis in lipedema tissue are regulated not only by chemical signals but also by mechanical forces. Increased interstitial fluid and a stiffening ECM create abnormal mechanical stress on adipocytes and fibroblasts. These mechanical stimuli are transmitted into the cell via integrins and lead to the activation of transcription cofactors such as Yes-associated protein (YAP) and transcriptional coactivator with PDZ-binding motif (TAZ), components of the Hippo pathway. Active YAP/TAZ enter the nucleus and alter gene expression; they promote adipogenic and fibrogenic gene programs, increase proliferation and inhibit apoptosis. This mechanotransduction process may play a critical role in the progressive nature of lipedema and its resistance to weight loss. Therefore, YAP/TAZ signal inhibition offers a new therapeutic strategy for targeting tissue stiffness and pathological fat accumulation in lipedema. This mechanism could explain the relationship between increased tissue stiffness and heightened nociceptor sensitivity [108].

In lipedema, intratissue pressure typically increases due to lymphatic insufficiency, leading to fluid retention, which causes patients to have marked tenderness upon palpation [109]. At the microvascular level, increased capillary permeability and perivascular inflammation lead to interstitial fluid accumulation, triggering the formation of intertissue edema. The excessive growth of adipose tissue in lipedema patients points to a histopathological change process supported by localized inflammatory processes. Particularly, microvascular leakage and impaired lymphatic drainage are considered the main factors triggering this process. These mechanisms lead to significant edema, a feeling of stiffness and pain in the legs of lipedema patients [110]. One study reported that skin sodium content is increased in lipedema patients and suggested this increase is a sign of the inflammation thought to be responsible for pain in lipedema [111].

In individuals diagnosed with lipedema, the hands and feet are usually spared and tenderness is observed upon palpation in the affected areas, whereas such distinct pain or tenderness is not present in obesity [112]. One of the most distinctive clinical features of lipedema is a finding called the “cuff phenomenon,” characterized by a marked narrowing in the ankle region. This finding is considered an important clinical criterion for differentiating lipedema from obesity and guides the diagnostic process. This condition, unlike in obesity, causes a sharp demarcation around the ankles and leads to a characteristic distribution of edema along the lower extremity [113]. It is also regarded as an important clinical feature that can be used for differential diagnosis from diseases such as lymphedema [114].

In contrast to obesity, it is reported that significant weight loss is mostly not achieved with diet and exercise in individuals diagnosed with lipedema and these patients generally have difficulty losing weight. Lipedema is characterized by marked inflammation and microvascular disorders in adipose tissue, resulting in a clinical picture distinct from obesity [104]. However, while lipedema can be controlled with early diagnosis and correct management, obesity usually requires a long-term, multidisciplinary treatment approach [105]. One of the most important factors in distinguishing it from obesity is that fat distribution in lipedema patients is usually limited to the lower extremities and does not show a significant reduction with weight loss. Sensory symptoms such as easy bruising, spontaneous pain, subcutaneous nodular structures and touch sensitivity are commonly seen in lipedema patients [100]. These sensory signs can provide important clues for differentiating lipedema from obesity. Obesity, on the other hand, is typically characterized by orthopedic pain due to physical load and symptoms related to metabolic complications [115].

Targeted treatment strategies developed based on the biology of lipedema are multidimensional. TLR4 antagonism, complement inhibitors and mast cell stabilizers carry the potential to suppress the proinflammatory response [116]. Modulation of the VEGF-C/VEGFR3 pathway is promising for supporting lymphatic function. Antifibrotic agents aimed at limiting ECM remodeling are under investigation. Hormone-based approaches considering estrogen receptor modulation and aromatase inhibitors are being evaluated [117,118,119,120]. Additionally, neuromodulatory strategies such as TRPV1 and TRPA1 antagonism [121] or sodium channel blockade hold potential for pain control [122]. In clinical practice, the biological effects of compression therapy, manual lymphatic drainage and tumescent liposuction are being assessed on a mechanistic level.

Beyond these biologically focused strategies, physical therapy and rehabilitative interventions function not just as supplements but as active modulators of physiological mechanisms in the treatment of lipedema. Consistent workout routines improve the efficiency of the skeletal muscle pump, which facilitates better microvascular flow and lymphatic transit, ultimately lowering the buildup of fluid in the extracellular spaces [123]. Muscle-derived signaling molecules (such as irisin and the anti-inflammatory form of IL-6) released during activity help to inhibit persistent low-grade inflammation; moreover, heightened activity of endothelial nitric oxide synthase can stabilize vascular integrity, decreasing the susceptibility of capillaries to damage [124,125]. Strength and stability training expand muscle volume, which increases the resting metabolic rate and, by triggering AMP-Activated Protein Kinase (AMPK) pathways, promotes the breakdown of fatty acids while hindering the formation of new lipids [126,127,128]. Such biological changes may play a role in curbing the excessive enlargement of fat cells within tissues affected by lipedema. Additionally, physical activity can modulate PPARγ signaling systems [129], thereby controlling the process of adipocyte development.

Physiotherapy applications affect not only circulation but also the structural integrity of tissue [130,131]. Manual lymphatic drainage and compression therapies regulate interstitial pressure to facilitate fluid movement [132]. Repeated mechanical stimuli can modulate fibroblast function and ECM remodeling [133]. This process may promote the realignment of collagen fibers, reducing tissue stiffness and limiting the hyperexcitability of nociceptive endings. Another significant effect of exercise and rehabilitation occurs at the central level. Aerobic exercise and functional activities increase Brain-Derived Neurotrophic Factor (BDNF) levels, supporting neuroplasticity and alleviating central sensitization [134,135]. Thus, the chronic pain frequently seen in lipedema can be controlled not only at the symptom level but also through modulation of the underlying neuroimmune mechanisms.

The effects of physiotherapy and exercise on molecular mechanisms in lipedema are summarized in Table 3.

The psychosocial effects of lipedema should not be overlooked. Due to esthetic concerns and pain, patients’ participation in social life may be restricted and psychological problems such as depression and anxiety may develop. Although similar psychosocial effects are observed in obesity [136,137], the appearance difference caused by lipedema and the lack of response to treatment can make the loss of self-confidence more pronounced in individuals [138]. Genetic factors play a role in the development of lipedema and some of these genes are also associated with brain function. For example, neuronal growth regulator 1 (NEGR1) genes have been shown to affect both adipose tissue and brain activities. These genetic links reveal that lipedema not only is a physical disease but also has neurological and psychological dimensions [47]. In this context, patients receiving psychological support and the prevention of social isolation are important factors for the success of the treatment process [138]. 

The proposed molecular effects of physiotherapy are largely derived from studies in other chronic inflammatory conditions or from preclinical models; direct evidence in lipedema is limited and requires further investigation [53].

Lipedema is increasingly understood as a medically complex condition rooted in a multifaceted pathophysiology, rather than a simple cosmetic concern. Its development involves an interplay of factors including microvascular inflammation, hormonal shifts, lymphatic system impairment and connective tissue abnormalities [139]. Central to this process is a state of chronic inflammation, characterized by elevated levels of proinflammatory cytokines such as IL-6, TNF-α and monocyte chemoattractant protein 1 (MCP-1). These mediators disrupt normal fat cell development, driving the pathological growth of adipose tissue and the recruitment of inflammatory cells. This process can be further complicated by secondary lymphatic damage and protein accumulation, particularly in cases where lipedema coexists with lymphedema [93]. For many patients, one of the most significant outcomes of this underlying biological process is ongoing, chronic pain. This symptom is commonly reported and often has neuropathic characteristics. The pain, along with increased sensitivity, is believed to result from persistent inflammation and mechanical strain affecting nearby nerve endings [140]. In addition, elevated pressure within the affected tissue may excessively stimulate sensory receptors, giving rise to symptoms such as allodynia and hyperesthesia [141].

In addition to the pathways discussed above, emerging evidence highlights the role of epigenetic regulation via microRNAs (miRNAs) in lipedema pathogenesis. A recent study by Cione et al. [142] profiled miRNA expression in lipedema adipose tissue and identified differentially expressed miRNAs that target genes involved in fundamental cellular processes, including cell cycle regulation, inflammatory pathways, endocrine resistance, and insulin signaling. Notably, some of these miRNAs were found to affect key molecules in estrogen signaling and angiogenesis. These findings provide new insights into the epigenetic regulation of lipedema and suggest that miRNAs may serve as future biomarkers or therapeutic targets. Furthermore, the statement that diet and exercise are entirely ineffective in lipedema requires qualification. While conventional low-calorie diets and general exercise programs often fail to reduce lipedematous fat deposits, emerging evidence suggests that specific dietary and exercise approaches may provide symptomatic benefits. Regarding exercise, although no large-scale controlled trials exist specifically for lipedema, appropriate physical activity (e.g., swimming, walking, resistance training) may improve lymphatic flow, reduce inflammation, and alleviate pain through muscle pump activation and myokine release, as discussed in Table 3. Regarding diet, several recent studies have demonstrated that a ketogenic diet can reduce pain and bruising, and improve quality of life in lipedema patients, likely by decreasing systemic inflammation [143,144,145,146,147,148]. One study also reported benefits with a low-carbohydrate diet [144]. These dietary approaches do not necessarily eliminate lipedematous fat but may manage associated symptoms. Therefore, while diet and exercise alone are insufficient to reverse lipedema-specific fat accumulation, they should not be dismissed as entirely ineffective; rather, they may serve as valuable adjunctive strategies for symptom control and overall metabolic health.

## 7. Conclusions and Future Directions

This research aimed to clarify the distinctions between obesity and lipedema by exploring their definitions, causes, clinical symptoms, underlying mechanisms and treatment strategies. It also investigated the sensory impact of both conditions, presenting current insights into the pain, sensitivity and inflammation processes patients experience. Ultimately, the goal was to compile a practical resource to guide healthcare professionals. Accurately differentiating between lipedema and obesity is crucial for early detection and for determining the most effective treatment path. 

Lipedema is a heterogeneous condition with variable clinical presentation, and diagnostic uncertainty remains common. The multifactorial nature of the disease—involving genetic predisposition (e.g., FOXC2, PROX1), hormonal influences, inflammatory profiles, and mechanical factors—contributes to this heterogeneity. Overlap with obesity, lymphedema, and other adipose disorders further complicates diagnosis. Therefore, the hypotheses and mechanisms discussed in this review require validation through well-designed clinical and translational studies. Until such evidence emerges, clinical diagnosis should remain multidisciplinary and individualized.

## Figures and Tables

**Table 1:** Biomarker profiles in lipedema and obesity.

Biomarker	Lipedema	Obesity	Distinguishing Feature	Clinical Significance	Level of Evidence
IL-6, TNF-α	Mild increase	Markedly elevated	Lower grade inflammation in lipedema	Aids in differential diagnosis; indicates severity of inflammation	Clinical, non-specific
Adiponectin	Relatively preserved	Low	Preservation of metabolic profile in lipedema	May indicate a lower risk of metabolic syndrome	Clinical (observational)
Leptin	Increased	More markedly increased	Increase in both, but more pronounced in obesity	Indicates adipose tissue dysfunction; not sufficient for diagnosis alone	Clinical
Complement (C3, C4)	Moderate increase	Strong increase	Immune activation is more limited in lipedema	Reflects immunological activity; supportive in differentiation from obesity	Clinical (exploratory)
Hyaluronan	Increased	Not significant	ECM remodeling is characteristic in lipedema	Can provide information about fibrosis and tissue stiffness	Preclinical/exploratory

Abbreviations: IL-6: Interleukin-6; TNF-α: Tumor Necrosis Factor-alpha; ECM: Extracellular Matrix. Level of evidence definitions: clinical = derived from human studies (case–control, cohort); preclinical = derived from animal or cell-based studies; exploratory = hypothesis-generating, not yet validated. Most biomarkers in this table lack specificity and require further validation.

**Table 2:** Clinical comparison of lipedema and obesity.

Feature	Lipedema	Obesity
Affected Areas	Typically lower extremities (legs, hips); hands and feet spared	Can occur throughout the body; often prominent in abdominal and upper body regions
Fat Distribution	Symmetrical, well-demarcated fat accumulation	Generally widespread and disproportionate fat distribution
Pain & Sensitivity	Tenderness and spontaneous pain are common	Pain is usually absent; if present, it is often joint- or mechanically related
Edema/Swelling	May have increasing edema during the day; lymphatic circulation may be affected	Edema is generally not seen, but may develop in advanced obesity due to venous insufficiency
Influencing Factors	Hormonal changes; genetic predisposition	Caloric intake, physical inactivity, genetic factors
Metabolic Complication	Rare	Frequent (diabetes mellitus, hypertension, etc.)
Disease Course	Progressive; becomes more pronounced with weight gain	Worsens with weight gain, but significant improvement is possible with weight loss
Response to Treatment	No significant improvement with diet and exercise alone	Weight loss is possible with diet, exercise and lifestyle changes
Treatment Methods	CDT, surgery (liposuction)	Diet, exercise, pharmacotherapy, surgery (bariatric)
Psychosocial Effects	Loss of self-confidence, depression and social isolation are common	Psychosocial effects may occur but can improve with weight control
Skin Changes	Bruising, subcutaneous nodules and tenderness may be seen	Skin usually shows no significant change, but striae may form with excessive weight gain
Exercise Tolerance	Pain and sensitivity may occur during physical activity	Generally low but can improve with weight loss

Abbreviations: CDT: Complete Decongestive Therapy.

**Table 3:** Potential effects of physiotherapy and exercise on molecular mechanisms in lipedema.

Exercise/Modality	Molecular Mechanism	Biological Effect	Clinical Correlation	Evidence Level (in Lipedema)
Aerobic Exercise (walking, cycling, swimming)	↑ BDNF, ↑ eNOS, ↓ proinflammatory cytokines (IL-6, TNF-α)	May enhance neuroplasticity, reduce vascular permeability, and suppress inflammation.	Reduction in central sensitization, decrease in pain intensity	Extrapolated (indirect)
Resistance Exercises	↑ AMPK activation, ↓ lipogenesis, ↑ fatty acid oxidation	May limit adipocyte hypertrophy and contribute to an increase in muscle mass.	Improvement in body composition, increased functional capacity	Preclinical/extrapolated
Balance & Functional Exercises	Modulated PPARγ signaling, myokine release (e.g., irisin)	May regulate adipogenesis balance and suppress low-grade inflammation.	Reduction in fatigue, improvement in quality of life	Hypothesis-generating
Manual Lymphatic Drainage	Regulation of fibroblast function and ECM remodeling	May promote collagen fiber realignment and reduce tissue stiffness.	Reduction in edema, decrease in palpation sensitivity	Clinical (limited)
Compression Therapy	Regulation of interstitial pressure, ↓ vascular permeability	May support microcirculation and reduce fluid accumulation.	Reduction in limb circumference, increased functional mobility	Clinical
Multimodal Exercise Programs (Combined)	Suppression of systemic inflammation, ↑ anti-inflammatory myokines	May contribute to metabolic stability and help balance the immune response.	Reduction in fatigue and pain, improvement in general health	Extrapolated

Abbreviations: BDNF: Brain-Derived Neurotrophic Factor; eNOS: Endothelial Nitric Oxide Synthase; IL-6: Interleukin-6; TNF-α: Tumor Necrosis Factor-alpha; AMPK: AMP-Activated Protein Kinase; PPARγ: Peroxisome Proliferator-Activated Receptor gamma; ECM: Extracellular Matrix. ↑ indicates increase; ↓ indicates decrease. Evidence levels: clinical = studies in lipedema patients exist; preclinical = animal/cell studies; extrapolated = derived from other disease models or basic science; hypothesis-generating = proposed mechanism without direct evidence. Most effects in this table are extrapolated or hypothesis-generating.

## Data Availability

No new data were created or analyzed in this study. Data sharing is not applicable to this article.

## Abbreviations

The following abbreviations are used in this manuscript:

AMPK	AMP-activated protein kinase
BMI	Body mass index
BDNF	Brain-derived neurotrophic factor
C/EBP	CCAAT/enhancer-binding protein
CDT	Complete decongestive therapy
CGRP	Calcitonin gene-related peptide
ECM	Extracellular matrix
eNOS	Endothelial nitric oxide synthase
ERα	Estrogen receptor alpha
ERβ	Estrogen receptor beta
EU	European Union
FOXC2	Forkhead box C2
GALT	Gut-associated lymphoid tissue
gfWAT	Gluteofemoral white adipose tissue
IL-1β	Interleukin-1 beta
IL-6	Interleukin-6
IL-18	Interleukin-18
LPL	Lipoprotein lipase
LPS	Lipopolysaccharide
MCP-1	Monocyte chemoattractant protein-1
MMP	Matrix metalloproteinase
MyD88	Myeloid differentiation primary response 88
NEGR1	Neuronal growth regulator 1
NF-κB	Nuclear factor kappa-B
NMDA	N-Methyl-D-Aspartate
OECD	Organisation for Economic Cooperation and Development
PPARγ	Peroxisome proliferator-activated receptor gamma
PROX1	Prospero homeobox 1
SCD1	Stearoyl-CoA desaturase-1
TAZ	Transcriptional coactivator with PDZ-binding motif
TIMP	Tissue inhibitor of metalloproteinase
TLR4	Toll-like receptor 4
TNF-α	Tumor necrosis factor-alpha
TRPA1	Transient receptor potential ankyrin 1
TRPV1	Transient receptor potential vanilloid 1
VEGF	Vascular endothelial growth factor
VEGF-C	Vascular endothelial growth factor -C
VEGFR3	Vascular endothelial growth factor receptor 3
WHO	World Health Organization
YAP	Yes-associated protein
